# Sudden cardiac death in synucleinopathies

**DOI:** 10.1093/jnen/nlad001

**Published:** 2023-01-20

**Authors:** Keivan Javanshiri, Tove Drakenberg, Mattias Haglund, Elisabet Englund

**Affiliations:** Division of Pathology, Department of Clinical Sciences Lund, Lund University, Lund, Sweden; Division of Pathology, Department of Clinical Sciences Lund, Lund University, Lund, Sweden; Division of Pathology, Department of Clinical Sciences Lund, Lund University, Lund, Sweden; Division of Pathology, Department of Clinical Sciences Lund, Lund University, Lund, Sweden

**Keywords:** α-Synuclein, Cause of death, Epicardial nerves, Lewy body disease, Multiple system atrophy, Parkinson disease, Synucleinopathies

## Abstract

The purpose of this study was to investigate the cause of death in subjects with α-synucleinopathies (ASs) and the confirmed presence of cardiac α-synuclein (α-syn), compared to non-AS disorders in a neuropathologically confirmed cohort. In total, 78 neuropathologically confirmed AS cases positive for cardiac α-syn were included in the study. Individuals with other neurocognitive diseases, having no α-syn in the brainstem or above, nor in cardiac nerves, served as controls (n = 53). Data regarding the cause of death, cardiac α-syn, pathological cardiac findings, and cardio- and cerebrovascular disease were assembled from autopsy reports and medical records. In the AS group, there was a significantly higher prevalence of sudden cardiac death ([SCD]; n = 40, 51.3%) compared to the control group (n = 12, 22.6%, p < 0.001). No statistically significant differences between the groups were reported regarding other cardiac conditions on autopsy or regarding cardio- and cerebrovascular disease from the medical records. The most prevalent cause of death in the AS group was SCD, which differed significantly from the control group. This suggests that α-syn deposits in cardiac nerves may cause lethal alterations in cardiac function, warranting further research.

## INTRODUCTION

Pathologically aggregated α-synuclein (α-syn) in the central nervous system (CNS) serves as the hallmark for α-synucleinopathies (ASs) (1), a disease group that comprises Lewy body disease (LBD) and multiple system atrophy (MSA), as well as clinically cognitively unimpaired individuals with α-syn in the brainstem (1, 2). LBD includes Parkinson disease (PD) and dementia with Lewy bodies (DLB) (1, 2).

AS is most likely a systemic disease involving the entire nervous system and not only limited to the CNS, as studies have found the aggregated protein in several organs, including the heart, gastrointestinal tract, and skin (3–7). Additionally, studies report the presence of cardiac α-syn in presymptomatic and early-stage PD patients as well as cases with only minimal α-syn pathology in the brainstem (5, 8–10). The clinical relevance of cardiac α-syn pathology in AS, however, is not established. It is believed to be associated with cardiac sympathetic denervation, which has been observed in LBD. Together with extra-cardiac noradrenergic denervation and arterial baroreflex failure, it may provoke autonomic dysfunction of the cardiovascular system, including orthostatic hypotension (OH) (11, 12). Interestingly, sympathetic denervation does not seem to appear as frequently in MSA, although α-syn pathology is present in the sympathetic neurons (12).

There has been research elucidating the possibility of cardiac α-syn as a contributing factor for sudden deaths in AS patients. It has been proposed to cause terminal arrhythmias in PD patients, with prolongation of the QTc interval as a potential underlying mechanism (13–15). A recent autopsy study found that a frequent cause of death among cases with DLB and MSA was circulatory failure, which differed from the other neurocognitive disorders (NCD) studied, that is, Alzheimer disease (AD), vascular dementia (VaD), frontotemporal lobar degeneration (FTLD), and cognitively healthy controls (16).

A study on relative survival in LBD found that the mortality in the patient group is more than 3 times higher during a 10-year follow-up when compared with the general population (17). In MSA, the median survival time is 7–9 years (18, 19). Consequently, AS patients have an increased mortality risk compared to the general population, and DLB has also been reported to have a higher mortality rate than AD (20, 21).

Thus, AS patients have a confirmed presence of cardiac α-syn pathology and a shortened expected lifespan (3, 17, 21). This demands additional research on the effects of cardiac α-syn. In this field, there is a scarcity of studies, including both clinical and pathological variables with neuropathologically confirmed diagnosis.

The aims of this study were to investigate a possible association between neuropathologically confirmed AS with cardiac α-syn, pathological cardiac findings, and cause of death and to evaluate the prevalence of clinical cardio- and cerebrovascular disease and risk factors in this patient group.

## MATERIALS AND METHODS

### Study design

We analyzed the immediate cause of death in deceased patients who were neuropathologically diagnosed with AS and with a confirmed manifestation of cardiac α-syn. An age-matched group with non-AS neurodegenerative or vascular brain disease and without α-syn in the cardiac nerves or in the brainstem or above, served as controls. Cases evaluated for inclusion were referred to the Clinical Department of Pathology in Lund, Sweden, between 2010 and August 2022; cases between 2010 and May 2021 were analyzed retrospectively, and cases between May 2021 and August 2022 were collected prospectively with regard to cardiac tissue sampling. The cause for referral for autopsy varied among individuals, from determining the cause of death to follow-up on specific diseases such as known cardiac disease or a tumor; there was not always an indication of the presence of known or suspected AS. Autopsy cardiovascular data and cause of death were extracted from the database systems LabVantage LIMS and SymPathy as well as pertinent clinical data on cardio- and cerebrovascular disease, risk factors such as hypertension (HT), type 2 diabetes mellitus (T2DM), and OH that were reported in the electronic health records and the Swedish National Diabetes Register (NDR), Gothenburg Sweden.

An approval from the Regional Ethical Review Board, Lund University, now The Swedish Ethical Review Authority, was obtained for this study, Nos 631-2015, 944-2017, 00051-2019, and 06582-2019, and from the NDR, RS 2022-01117.

### Study subjects

All deceased patients with neuropathologically confirmed AS registered from 2010 and onwards were evaluated for inclusion (n = 101). All individuals underwent a regular clinical whole-body autopsy procedure with the subsequent neuropathologic investigation. Subjects were excluded due to limited neuropathological examination and/or insufficiently sampled cardiac tissue void of epicardial surface with stainable nerves.

The neuropathological diagnosis of AS has been described in detail in previous work (5). LBD pathology connotes cytoplasmic α-syn inclusions; Lewy bodies (LBs), the small elongated neuritic α-syn aggregates; Lewy neurites (LNs), and in MSA glial inclusions of the misfolded protein (1, 2). Disease staging was based on the neuroanatomical distribution of α-syn, where LBD was subdivided into 3 groups: LBD brainstem, LBD limbic, and LBD cortical (22). MSA was further classified as MSA-C (cerebellar), MSA-P (Parkinsonian), or MSA-C+P (mixed pathology) (23). The presence of α-syn pathology in the brainstem or above was a requirement, although several subjects had a concomitant and more dominant pathology of other major NCD: AD, VaD, FTLD, or admixtures of these.

On referral to autopsy, most AS cases had a history of suspected or verified clinical NCD. Among the 101 subjects reviewed for inclusion, 78 AS subjects with cardiac α-syn were included. For demographic data on the study group, see Table 1. Seventy-four (95%) had LBD and 4 (5%) had MSA. Within the LBD group, 39 had cortical LBD, 11 had limbic LBD, and 24 had brainstem LBD (Table 2). Among these, 29 cases had concomitant pathology of other NCD, where most cases had a concurrent AD pathology, n = 9; see Table 2. One of the cases with MSA could not be specified further due to the limited amount of brain tissue collected for the neuropathological analysis. The other 3 cases included 2 individuals with MSA-C and 1 with MSA-P (Table 2).

**Table 1.** Demographic data for all subjects

Diagnoses	Subjects, n (%)	Sex, n (%) Men	Age at death*
Total	131 (100.0)	85 (64.9)	78.0 (10.0)
AS group	78 (59.5)	55 (70.5)	77.0 (9.0)
Control group	53 (40.5)	30 (56.6)	78.5 (10.5)

AS, α-synucleinopathy.

*Presented in median years, interquartile range within brackets.

**Table 2.** Neuropathological diagnoses for all subjects

Neuropathological diagnosis	n (%)
AS group	78 (100.0)
LBD	74 (94.9)
LBD brainstem	24
LBD limbic	11
LBD cortical	39
MSA	4 (5.1)
MSA*	1
MSA-P	1
MSA-C	2
Neuropathological comorbidity^†^	29 (37.2)
AD	9
VaD	8
FTLD	5
Mixed pathologies^‡^	7
Control group	53 (100.0)
AD	12 (22.6)
VaD	11 (20.8)
FTLD	18 (34.0)
Mixed pathologies^¶^	12 (22.6)

AS, α-synucleinopathy; LBD, Lewy body disease; MSA, multiple system atrophy; MSA-C, MSA cerebellar; MSA-P, MSA parkinsonian; AD, Alzheimer disease; VaD, vascular dementia; FTLD, frontotemporal lobar degeneration; including cases with FTLD-Tau, FTLD-TDP43, progressive supranuclear palsy and corticobasal degeneration.

*MSA not further specified.

^†^Neuropathological comorbidity within the AS group.

^‡^Including AD, VaD, and FTLD, of which 5 had a combination of AD + VaD.

^¶^Including AD, VaD, and FTLD, of which 8 had a combination of AD + VaD.

### Control subjects

The control group consisted of an age-matched group of 53 subjects with non-AS neurodegenerative or vascular pathology, without α-syn in the brainstem and above, and with accessible heart tissue including cardiac nerves that were negative for α-syn (Table 1). All controls had been diagnosed clinically with or had a suspected NCD and were referred for autopsy during the same period as the AS group. The neuropathological diagnoses in the control group consisted of 12 cases (22.6%) with AD pathology, 11 cases (20.8%) with VaD pathology, 18 cases (34.0%) with FTLD pathology, and 12 cases (22.6%) with mixed pathologies (Table 2). In the latter group, a majority had a combination of AD and VaD pathology (n = 8).

### Cardiac α-syn pathology

**Figure 1.**
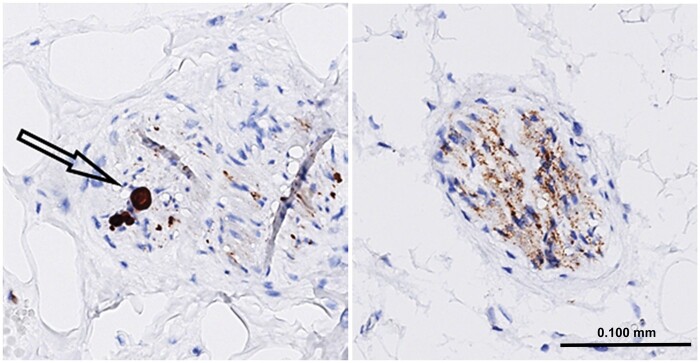
Cardiac epicardial nerves strongly positive for α-synuclein pathology. Lewy body marked with an arrow. α-Synuclein immunohistochemistry with hematoxylin counterstain.

A detailed description of the pathological work and subsequent immunohistochemical analysis following autopsy has been described in a previous study (5). Regarding the retrospectively analyzed cases, myocardial sections were stained with hematoxylin and eosin and subsequently analyzed for selection of the section most clearly harboring nerves. The prospectively collected cases were sampled from the epicardium and the selected tissue block was sectioned and stained with hematoxylin and eosin and α-syn at one occasion. A corresponding tissue section was stained with a monoclonal mouse anti-α-syn concentrate antibody (Invitrogen by Life Technologies, Synuclein clone LB509, Lot no. 2068148) to visualize α-syn deposits. The same α-syn antibody was applied in the brain sections during the neuropathologic diagnostic workup. Cardiac α-syn pathology was considered present when there were α-syn deposits (granular or filamentous positivities or LBs) within 1 or multiple cardiac nerves (Fig. 1).

### Data from autopsy reports

Examination of the heart and arterial system was done in accordance with routine autopsy protocols within the department; all pathologists used similar terms for reporting abnormal findings. The following data on each study subject were extracted from the autopsy reports: underlying and immediate cause of death, cardiac hypertrophy, cardiac fibrosis, cardiac valve pathology, nephrosclerosis, and coronary and aortic atherosclerosis.

The cause of death was defined through extensive analyses of all findings from an autopsy, clinical medical history, and information regarding the sequence of events preceding death. In numerous cases, no obvious immediate cause of death was reported on autopsy, and sudden cardiac death (SCD) was suspected. These cases were reanalyzed through reading of the autopsy reports and analysis of the heart tissue samples by light microscopy. The certainty of the cause of death upon SCD was evaluated according to Basso et al (24), and an attempt was made to estimate whether the SCDs were due to a cardiac disease or other causes (25, 26). Potential genetic examination and toxicology testing were considered if presumed and possible in the prospectively sampled cases. Additionally, through the autopsy reports, an evaluation of arteriosclerosis in the coronary arteries and other cardiac pathology was made from data in the autopsy reports to assess whether the SCDs were related to coronary or noncoronary reasons (27, 28).

Cardiac hypertrophy was counted as positive when mentioned in the autopsy report or when the weight of the heart was ≥500 g. Cardiac fibrosis was defined as positive if there were findings of moderate to severe fibrosis in the myocardium. Mild findings of solitary fibrosis in the papillary muscles were not noted. Cardiac valve disease was defined as observed valve pathology on autopsy or when noted in the clinical reports.

To evaluate the prevalence of atherosclerosis in the coronary arteries and the aorta, the assessment from an earlier study was slightly modified (29). The reported severity degree in each subject was converted into a corresponding number according to the scales below. A 2-degree scale was used for the coronary arteries since their sclerosis degrees were mainly described as “mild” or “marked,” thereby translated to no-to-mild or moderate-to-severe sclerosis, respectively. On the contrary, the aortas were generally described to exhibit a wider variety of sclerosis severity, therefore, a 3-degree scale was used.

The following grading system for atherosclerosis in the coronary arteries was used: 0: no-to-mild sclerosis, with mildly stenosed arterial lumen, and 1: moderate-to-severe sclerosis, with complicated plaques and/or considerable narrowing of vessel lumen in at least 1 location.

For atherosclerosis in the aorta, we used: 0: no-to-mild sclerosis, with small areas of fibrous plaques; 1: moderate sclerosis, with patchy areas of fibrous and complicated plaques; and 2: severe sclerosis, with complicated or ulcerated plaques and/or mentioning of dissection or aneurysm.

### Data from clinical records

The medical records and the NDR were used to assemble information regarding demographics, clinical cardio- and cerebrovascular disease, and risk factors (HT and T2DM). The following data were collected: sex, age of death, congestive heart failure, arrhythmia, history of coronary artery disease/angina pectoris, history of myocardial infarction, history of stroke or transient ischemic attack, electrocardiogram (ECG) for acquiring QTc, HT, T2DM, and OH.

QTc was calculated based on Framingham correction (30, 31). A QTc >450 ms was considered prolonged for men and >460 ms for women (30). Only the last ECG tracing was analyzed, and in a total of 131 subjects, 23 were excluded because of findings compatible with acute ischemia, rhythm aberrations, or missing ECG tracings in the medical journals. Furthermore, cases with a normal QTc were excluded if the time between the ECG tracing and death was >3 years (n = 24). All cases with a prolonged QTc on the last recorded ECG were included regardless of the time between the ECG tracing and the time of death. Thus, a total of 84 cases were suitable for analysis, 50 in the AS group and 34 in the control group.

Subjects with a documented diagnosis of HT or antihypertensive treatment were considered positive for HT. Only the presence or absence of a T2DM diagnosis was considered, and not the choice of treatment or disease duration.

Cases were considered positive for the variable “clinical heart disease” if positive for ≥1 of the following: congestive heart failure, arrhythmia, history of coronary artery disease/angina pectoris, or previous myocardial infarction. The subjects were assumed to be free of all studied parameters, including cardiovascular disease and/or symptoms and risk factors if no documentation regarding these conditions was found.

### Data analysis

Statistical analyses were conducted using Microsoft Excel and IBM SPSS Statistics 28. Pearson chi-squared test was used for comparison between the groups. Cause of death and the clinicopathological parameters were compared pairwise and not as a group. QTc values were dichotomized into prolonged and normal values and compared dichotomously. A p value of ≤0.05 was considered statistically significant. A subanalysis was performed where the subjects with LBD brainstem were extracted and compared to the remainder of the AS group (limbic, cortical, and MSA) and the control group. In this case, the 3 groups were compared using Pearson chi-squared test; if a difference was found, the same analysis was performed separately comparing each group with the others. The Bonferroni correction was used to adjust the level of significance, and a significant p value was = 0.017, as 3 consecutive tests were performed. Regarding the evaluation of arterial sclerosis, no statistical tests were performed.

## RESULTS

### Immediate causes of death

The 3 most common immediate causes of death in the study group were SCD (n = 40, 51.3%), acute myocardial infarction (n = 21, 26.9%), and pulmonary embolism (n = 7, 9.0%). Among the control cases, the 3 most common causes were acute myocardial infarction (n = 22, 41.5%), SCD (n = 12, 22.6%), and pulmonary embolism (n = 7, 13.2%) (Table 3). The incidence of SCD was significantly higher in the study group compared to the control group (p < 0.001). There were no other statistically significant differences in the cause of death between the groups. In the subanalysis, the prevalence of SCD differed significantly in individuals with MSA, limbic LBD, or cortical LBD from the control group (p < 0.001) but not from the brainstem LBD group (p = 0.059). There was no difference between individuals with brainstem LBD and the control group (p = 0.179*).*

**Table 3.** Immediate causes of death for the AS group with cardiac α-synuclein deposits compared to the control group without cardiac α-synuclein deposits

Cause of death	AS group n (%)	Control group n (%)	p value^†^
Total	78 (100.0)	53 (100.0)	
SCD	40 (51.3)	12 (22.6)	<0.001
AMI	21 (26.9)	22 (41.5)	0.081
Pulmonary embolism	7 (9.0)	7 (13.2)	0.441
Aortic dissection	2 (2.6)	0 (0.0)	X^‡^
Stroke	1 (1.3)	2 (3.8)	X^‡^
Other*	7 (9.0)	10 (18.9)	X^‡^

AS, α-synucleinopathy; SCD, sudden cardiac death; AMI, acute myocardial infarction.

*Including cases with multi organ failure, malignancies, infection/septicemia, and asphyxia.

^†^Chi-square value between study group and control group comparing the cause of death pairwise. A p value ≤ 0.05 was considered statistically significant.

^‡^No statistical analysis was made due to few cases in each group.

In the cases in the AS group judged to have died from SCD, there were 9 cases with brainstem LBD, 7 cases with limbic LBD, 22 cases with cortical LBD, and 2 cases with MSA. Among these, 18 subjects had a combination of other pathologies. In the control group, the 12 SCDs were represented by 6 cases of FTLD, 5 cases of AD, and 1 case of mixed AD-VaD. They were all considered to have died from cardiac causes but for noncoronary reasons, that is, they had signs of terminal cardiac failure without attributes of ischemic heart disease such as plaque complications or discolored myocardium. Additionally, the SCD could not be explained by other cardiac pathology, such as heart valve disease, cardiomyopathy, hypertrophic or dilated myocardium, or viral infections found at autopsy.

Microscopically in the SCD cases, much of the analyzed myocardium looked devitalized, often with eosinophilic cardiomyocytes and reduced cell cohesiveness but with no focal pathology such as blurred cell borders from early cardiomyocyte dissolution or blood extravasation; nor were there other characteristic traits seen in acute myocardial infarction (32). Furthermore, there were no microthrombi in the vessels; instead, congestion was often found.

Pneumonia was the dominating underlying cause of death in both the study group and the control group, with a prevalence of 23 cases (29.5%) and 26 cases (49.1%), respectively.

### Parameters from autopsy reports

There was no significant difference between the AS and the non-AS control group with respect to the prevalence of cardiac pathology as reported from autopsy (Table 4). The subjects with brainstem LBD exhibited the same profile as the rest of the AS group. The only autopsy parameter that differed between the entire AS group and the control group was the presence and absence, respectively, of epicardial nerve synuclein pathology.

**Table 4.** Autopsy-reported variables in the AS group with cardiac α-synuclein deposits compared to the control group without cardiac α-synuclein deposits

Variables		AS group n (%)	Control group n (%)	p value*
Total		78 (100.0)	53 (100.0)	
Cardiac hypertrophy		44 (55.1)	30 (56.6)	0.867
Myocardial infarction		20 (25.6)	18 (34.0)	0.303
Cardiac fibrosis		38 (48.7)	26 (49.1)	0.970
Heart valve disease		17 (21.8)	9 (17.0)	0.498
Cardiac dilatation		13 (16.7)	7 (13.2)	0.589
Nephrosclerosis		35 (44.9)	26 (49.1)	0.637
Coronary sclerosis	None-mild	32 (41.0)	21 (39.6)	X
	Moderate-severe	46 (59.0)	32 (60.4)	
Aortic sclerosis	None-mild	13 (16.7)	9 (17.0)	X
	Moderate	26 (33.3)	21 (39.6)	
	Severe	39 (50.0)	23 (43.4)	

AS, α-synucleinopathy; α-syn, α-synuclein; X, no statistical analysis was made.

* Chi-square value between study group and control group. A p value ≤ 0.05 was considered statistically significant.

### Parameters from clinical records

The prevalence of OH differed statistically between the groups and was reported in 34 cases (43.6%) in the AS group and 6 cases (11.3%) in the control group (p ≤ 0.001). When subjects with brainstem LBD were analyzed as a separate group, OH was the only parameter that demonstrated a statistical difference among the 3 groups (p = 0.001) and after the Bonferroni correction between the brainstem LBD group and the rest of the AS group, with 5 (20.8%) and 29 (53.7%) cases, respectively (p = 0.007). No difference was observed regarding OH between the subjects with brainstem LBD and the control group (p = 0.269). Comparing the entire AS and the control groups (i.e. dying from various different causes), there were no additional differences regarding the prevalence of clinical cardio- or cerebrovascular disease, arrhythmia, HT, and T2DM (Table 5). Neither were there any differences in prolonged QTc, with 9 cases (18.0%) in the AS group and 5 cases (14.7%) in the control group (p = 0.691). The time between the ECG tracings and death was the same in the total cohort and the study groups (data not shown). Among the cases considered to have died from SCD, 7 had a prolonged QTc (i.e. 5 cases within the AS group and 2 cases in the control group [p = 0.656]), and 14 cases had a history of arrhythmia (ie 12 cases within the AS group and 2 cases in the control group [p = 0.475]).

**Table 5.** Clinical data assembled in the AS group with cardiac α-synuclein deposits compared to the control group without cardiac α-synuclein deposits

Clinical data	AS group n (%)	Control group n (%)	p value^‡^
Total	78 (100)	53 (100)	
Congestive heart failure	10 (12.8)	10 (18.9)	0.345
Arrhythmia	22 (28.2)	15 (28.3)	0.990
AF	15 (19.5)	12 (22.2)	X
Third-degree AV block	3 (3.9)	2 (3.7)	X
SSS	4 (5.2)	1 (1.9)	X
Prolonged QTc*	9 (18.0)	5 (14.7)	0.691
Coronary artery disease/angina pectoris	9 (11.5)	4 (7.5)	0.453
History of myocardial infarction	9 (11.5)	7 (13.2)	0.775
History of stroke and/or TIA	12 (15.4)	12 (22.6)	0.292
Orthostatic hypotension	34 (43.6)	6 (11.3)	<0.001
Hypertension	45 (57.7)	28 (52.8)	0.582
Diabetes	11 (14.1)	10 (18.9)	0.466
Clinical heart disease^†^	45 (57.7)	22 (41.5)	0.069

AS, α-synucleinopathy; AF, atrial fibrillation; AV block 3, atrioventricular block 3rd degree; SSS, sick sinus syndrome; TIA, transient ischemic attack; X, no statistical analysis was made.

*A total of 84 cases (of 131 cases in total) had ECG tracings eligible for analysis. Fifty cases in the AS group and 34 cases in the control group.

^†^Positive for ≥1 of the following: congestive heart failure, arrhythmia, coronary artery disease/angina pectoris, or previous myocardial infarction.

‡Chi-square value between study group and control group. A p value ≤0.05 was considered statistically significant.

## DISCUSSION

The aim of this study was to explore the potential effects of cardiac α-syn in AS by examining the cause of death, pathological cardiac findings, the prevalence of cardio- or cerebrovascular disease, and risk factors in affected individuals. Our main findings were: (1) a high incidence of SCD in the AS group, (2) a high presence of cardiac α-syn in epicardial nerves in all stages of LBD, and (3) an absence of differences in other autopsy-reported cardiac findings and clinical cardio- and cerebrovascular disease, HT, and T2DM.

The AS patients, who were all positive for cardiac α-syn, had similar degrees of “traditional vascular-ischemic” pathological cardiac conditions on autopsy. Thus, the likely main cause of death was SCD, to a greater extent than in the control subjects. Through extensive analysis of the cardiac samples, pathological findings, and the clinical findings, the SCDs were considered to be of cardiac origin but not related to ischemic burden. The rate of ischemic pathology did not differ between the groups and was associated mostly with VaD. There were no statistical differences regarding the prevalence of myocardial infarctions nor any numerical differences considering the degree of arteriosclerosis in the aorta and the coronary arteries. These findings raise the possibility that the previously reported high prevalence of circulatory failure as a cause of death in DLB and MSA (16) could be due to SCD. Potentially, α-syn could exert pathological effects on the heart, which are not easily detected through regular autopsy routines and may contribute to the cause of death. In view of the known negative effects of α-syn CNS functions, it is plausible to assume that they would apply to the peripheral nervous system as well (22, 33). Furthermore, the findings of statistically different prevalences of SCDs between the control group and individuals with MSA, limbic, or cortical LBD, but not brainstem LBD, suggests that there might be an increased burden of cardiac α-syn with disease progression in the brain. However, the results also indicate that even a limited brain pathology connotes the presence of cardiac α-syn pathology sufficient to provoke lethal alterations, as no differences were seen regarding the occurrence of SCDs between cases with brainstem LBD and cases with MSA, limbic, or cortical LBD.

In line with what is already proposed, a potential explanation for the cardiac deaths would be terminal arrhythmias due to disturbed cardiac nerve function caused by α-syn deposits (13, 14). It is noteworthy that 4 of the 78 study subjects had sick sinus syndrome, which is in contrast to the general age-matched population in which a prevalence of 1 in 600 has been reported (34). Furthermore, the prevalence of congestive heart failure and history of stroke and/or transient ischemic attack were both approximately twice as common in AS as in the general population described in the Swedish study (35). There are many explanations to the existence of these conditions, such as arteriosclerosis and HT. Even though we did not find any differences in prolonged QTc between the study groups, in light of the discussion above, the SCDs could be a consequence of arrhythmias (36).

We found cardiac α-syn in all studied MSA cases, a result that is dissimilar to other studies reporting a lower frequency of these aggregates and better-preserved innervation (3, 37). However, due to the small sample size (n = 4), no conclusions can be drawn on these findings.

The finding of a higher prevalence of OH in the AS group compared to the controls could be explained by the potential association between cardiac α-syn and sympathetic denervation, which might result in OH (11, 12, 38, 39), even if extra-cardiac sympathetic denervation would be expected to contribute in a similar way. A possible diagnostic tool to detect cardiac sympathetic denervation in PD and DLB as well as probable autonomic dysfunction is 123I-metaiodobenzylguanidine myocardial scintigraphy (40–42). This method could perhaps be used more frequently in diagnostic investigation to evaluate and confirm the cardiac burden in these patients. In our cohort, the method was performed in only a few cases and was not specifically reported.

Dysregulated blood pressure was high in the AS group (57.7%), which is confirmed in some studies (43, 44), but is in contrast to others (45). The high rate of HT could be explained by the cases with significant VaD pathology (n = 14, 18%) (29). However, only 44.9% had autopsy-confirmed nephrosclerosis, a consequence of HT, suggesting a relatively mild degree of hypertension in many individuals (46). Additionally, the prevalence of T2DM was low in the AS group (14.1%), which is in line with previous studies (45).

A strength of this study is the large sample size and the extensive neuropathological and cardiac examination to confirm the presence of α-syn, the SCDs, and the cause of death. The inclusion of subjects with brainstem LBD is a further strength, as these subjects do not display cognitive symptoms related to their synuclein pathology and might be overlooked or misjudged in other studies (47). Furthermore, our findings confirm that even cases with early Lewy body pathology, restricted to the brainstem, display aggregates of α-syn in their cardiac nerves. Additionally, the validation of α-syn pathology in cardiac nerves was performed by or controlled afterward by the same neuropathologist, thus increasing the consistency of the evaluations and the decision to include or exclude cases. Another strength is the broad investigation of pertinent clinical and pathological data portraying cardio- and cerebrovascular disease.

Limitations include the myocardial sampling of the retrospectively analyzed cases. This was not done with the current study in mind, and several of these subjects lacked stainable nerves, which are best studied in epicardial tissue (48). These samples were originally taken with the intention of verifying or refuting a myocardial infarction and, hence, from different locations in each sample, guided by a discolored myocardium. Consequently, 18 cases were excluded from the study, probably leading to fewer study subjects in the AS group. The suboptimal sampling was additionally confirmed by the fact that all prospectively analyzed cases, which were sampled from the epicardium with the current study in mind, were positive for α-syn. This suggests that all AS cases are likely to have α-syn deposits in their cardiac nerves. One may alternatively argue that the cases lacking stainable nerves could be the ones most severely affected due to the known cardiac sympathetic denervation in AS (11). However, we do not judge this to be a dominant feature, since the different AS groups, with or without clinical synuclein disease, appeared not to differ in terms of epicardial nerve engagement. Additionally, as the cardiac sampling was nonhomogenous, no conclusions could be drawn regarding whether a higher grading intensity or quantity of cardiac α-syn deposits was associated with the SCDs in these cases. Future studies should aim to sample from the epicardium as well as to investigate the distribution of cardiac α-syn to clarify whether all nerves or a specific locus of nerves is more commonly affected. The distribution of α-syn within the nerve tissue and among the different nerves in our cohort was irregular, which might suggest that not all nerves are affected.

## CONCLUSION

In conclusion, we found a high incidence of SCDs and the absence of other significant vascular-ischemic pathological cardiac findings, both clinically and on autopsy. This indicates that α-syn deposits in cardiac nerves may cause lethal pathological alterations in cardiac function. These findings highlight the need for further research of the cardiac involvement in AS and suggest that more extensive cardiac examinations of AS patients might be motivated.
